# Pilot Program to Improve Self-Management of Patients with Heart Failure by Redesigning Care Coordination

**DOI:** 10.1155/2014/836921

**Published:** 2014-04-23

**Authors:** Jessica D. Shaw, Daniel J. O'Neal, Kris Siddharthan, Britta I. Neugaard

**Affiliations:** ^1^James A. Haley Veterans' Hospital, 13000 Bruce B. Downs Boulevard, Tampa, FL 33612, USA; ^2^College of Public Health, University of South Florida, 13201 Bruce B. Downs Boulevard, Tampa, FL 33612, USA

## Abstract

*Objectives*. We tested both an educational and a care coordination element of health care to examine if better disease-specific knowledge leads to successful self-management of heart failure (HF). *Background*. The high utilization of health care resources and poor patient outcomes associated with HF justify tests of change to improve self-management of HF. *Methods*. This prospective study tested two components of the Chronic Care Model (clinical information systems and self-management support) to improve outcomes in the self-management of HF among patients who received intensive education and care coordination during their acute care stay. A postdischarge follow-up phone call assessed their knowledge of HF self-management compared to usual care patients. *Results*. There were 20 patients each in the intervention and usual care groups. Intervention patients were more likely to have a scale at home, write down their weight, and practice new or different health behaviors. *Conclusion*. Patients receiving more intensive education knew more about their disease and were better able to self-manage their weight compared to patients receiving standard care.

## 1. Introduction

Heart failure is a chronic disease resulting from multiple diseases of the heart such as coronary artery disease [1]. According to the American Heart Association (AHA), nearly 5.7 million people in the United States currently have heart failure (HF) [2]. After age 65 the incidence of heart failure approaches 10 per 1,000 population and 7.2% of deaths can be attributed to heart failure [3]. Heart failure is the most common principal discharge diagnosis among Medicare beneficiaries and half of the HF patients above 65 years old are readmitted within 6 months of hospital discharge [4]. Within the Veterans Health Administration (VHA) heart failure is a high cost disease. The VHA saw nearly 6 million unique veterans in 2011; 50% were 65 years or older and 9% were female. In the same year, 2007, 424 Veterans had a diagnosis of heart failure (ICD-9 428) and 20% of HF patients had an all-cause readmission within 30 days of discharge.

This evidence shows the continued and growing burden of HF in our society. The impact of HF can be seen physically, cognitively, emotionally, and socially, contributing to the multifaceted morbidity and mortality of the disease [5]. Among older adults, HF results in greater challenges in treatment options. Many older adults have multimorbid medical conditions, a complex medication list, limited or absent social support, and other factors limiting the success of some treatment options [6]. Additionally, HF patients have low levels of knowledge of their HF medications, including the purposes of weight and sodium management, and often do not recognize the correct definition of HF [7]. Also, patient comprehension of self-care for heart failure has been found to be low, including the need to weigh themselves on a regular basis and limit fluid intake [8]. Increased knowledge about HF is necessary to produce changes in health behavior and should, therefore, be a primary target for interventions [9]. Systematic HF education tailored to the patient should help patients synthesize information for HF self-management [10]. Recommendations for self-management include symptom recognition, fluid and sodium management, nutrition and weight management, smoking cessation, and physical activity [11]. Self-management teaching has been associated with reducing HF-related readmission rates [12]. Heart failure patients who exhibit more engagement in self-management were found to have fewer hospitalizations and all-cause mortality [13]. A RCT by Smeulders found that heart failure patients who received a nurse-led self-management program that included medical, social, and self-management skills demonstrated better cognitive symptom management and self-care behavior [14]. Another report found that use of multidisciplinary teams and in-person communication with patients/caregivers led to better outcomes with a reduction in both HF readmissions and the number of days hospitalized [15]. Additionally, a meta-analysis found that discharge planning and postdischarge support for heart failure patients reduced readmissions [16].

Despite the various types of interventions and treatment options available, long-term improvements in HF outcomes of patients have been slow [17]. These factors combined with the high level of utilization of heath care resources within the Veterans Health Administration (VHA) and the chronic, progressive nature of this disease signal the need for improved care coordination strategies to reduce the morbidity and mortality of HF. To address this, the present study looked at the institution's ability to identify, characterize, and support its population of HF patients and the patients' self-management of HF. The purpose of this study was to examine the association between enhanced care coordination interventions and patient self-management of HF.

## 2. Methods

### 2.1. Study Design

This was a prospective, pilot study conducted at a large Veteran's hospital located in the Southeastern United States. The study established a set of care-coordination strategies, achieved institutional support for their deployment, and provided a follow-up phone call to assess self-management of disease since hospital discharge.

### 2.2. Study Participants

The study inclusion criteria were patients admitted to any acute care unit who were being treated for HF. Patients were excluded if they did not consent to participate, were discharged to a nursing home or assisted living facility, did not have a telephone/cell phone, or were not able to provide valid informed consent. Based on the number of patients admitted for heart failure each month at the facility, it was estimated that 40 patients could realistically be enrolled during the study time frame for this pilot study.

### 2.3. Selection Procedures

Study staff included a cardiologist, registered nurses, research assistants, and health services researchers. The institution established a dashboard for patient administrative data to facilitate study staff daily review of a list of admitted patients to the Medicine Service with a diagnosis of heart failure to screen for potential study participants. The diagnosis of heart failure was made based on the clinical judgment of the attending physician of the admitting medical team. The patients' medical records were reviewed for the admitting diagnosis of heart failure. Patients with both systolic and preserved LV function were included in this study. The patients' progress notes (Interdisciplinary Patient Assessment) were reviewed to determine if the patient had any mental health or personality impairment; if so, they were excluded. Patients who met the inclusion criteria were approached by regular hospital staff to get permission to have study staff speak to them. Study staff then contacted the patient to determine their interest in participating in the study. Study staff assessed the patient and family as to whether or not the patient could provide valid informed consent. Patients who were not able to provide valid informed consent based on not being able to fully comprehend the study and what was being asked of them were not enrolled. Patients and their caregivers were given the opportunity to ask questions and review the study prior to enrollment. Informed consent was obtained for each study patient after study details were discussed. Those who agreed to participate were assigned to the intervention group if they were admitted to one of seven general medicine service attending teams or one of three cardiology attending teams. In the facility there are 3 cardiology attending teams (1 of which is only for observation patients) and 7 general medicine teams. The medicine teams rotate admissions based on random day-of-the-week assignment. Cardiology teams are similarly assigned to cover admissions according to random day-of-the week assignment. Patients with heart failure in the intervention or nonintervention group could have been patients seen by either cardiology or general medicine teams. All other patients being treated for HF on the remaining cardiology and medicine teams comprised the usual care group.

### 2.4. Intervention

The Chronic Care Model was used as the framework for the test of organizational change in this study. (Figure 3) [18]. Boyde's review of educational interventions for patients with heart failure was used to select an eclectic model for the educational intervention [19]. Study staff training in educational interventions used the seven studies from Boyde's review that used a theoretical model, as well as Redman's classic patient education text to prepare in such areas as: avoiding leading questions, use of wait time for responses, and assessing for health literacy and numeracy skills [20, 21]. The Chronic Care Model poses that there are several evidence-based interventions to improve processes of care: delivery system design, decision support, information systems, linkages to the community, self-management support, and organization of the health system. This model has been used in multiple areas for chronic disease management, including heart failure [22–24]. Applying this model, the emphasis of the intervention was providing patients with self-management support of their heart failure so that they would be empowered and prepared to manage their own health. Institutional commitment to sorting and ordering administrative data was a prerequisite for this study.

The model shows that productive interactions with an informed and activated patient and a prepared and proactive practice team lead to improved patient outcomes. Patients have a central role in managing their own health and the practice team is there to support the patient. Self-management has been defined by Richard and Shea [25] as the “ability of the individual, in conjunction with family, community, and healthcare professionals, to manage symptoms, treatments, lifestyle changes, and psychosocial, cultural, and spiritual consequences of health conditions.” The care team provides disease-specific information to the intervention patients and their partners to help them engage in self-management and make informed decisions about their health care. Intervention patients are instructed on how to self-monitor their condition so that as symptoms arise they know what actions need to be taken.

In this pilot study, specific care coordination efforts were delivered during the hospitalization to encourage patient self-management. Care coordination incorporated patient/family in care planning as inpatient and outpatient, provided lateral integration of services to reduce fragmentation of care, and streamlined the postdischarge process (see Table 1). Care coordination programs typically involve teaching patients about their self-care, medications, and how to communicate with their healthcare providers, as well as arranging for any health-related services needed. The intervention was delivered by RN patient care facilitators (RN-PCFs) who have roles similar to discharge planners or transition specialists. These RN-PCFs act as care coordinators who work to transition the patients to the next level of care. Two of these RN-PCFs were selected from a pool of 10 RN-PCFs as study nurses to provide intensive HF education to the intervened patients. They were selected because of their training and background. Both RN-PCFs had previous involvement in heart failure research studies and both held bachelor's degrees in nursing; one had a certificate in case management. Multiple educational materials including an enhanced education workbook (Krames' Go-to-Guide to Living Well with Heart Failure) and a refrigerator magnet with key signs and symptoms of early to late HF were used in educating patients on their disease. The Krames' workbook was evaluated as a print aid for staff and patient/family training. It was used as an education material based on past positive feedback on the product. Krames applies the Patient Engagement Framework to inform, engage, and empower the patient, while partnering with them to coordinate their care. The workbook included information on lifestyle, diet, and pharmacy. The nurses educated patients on changes in symptoms in order for the patient to understand how to self-monitor their weight and other symptoms related to heart failure. Study nurses reviewed the HF education workbooks with the intervention patients and their family or caregivers. Workbooks had logs for patients to record their weight, blood pressure readings, medications, and sodium intake. They also included an enhanced education packet with nutrition guidelines. Refrigerator magnets were provided to increase patient recognition of HF symptoms (Figure 2). Study nurses educated the patient on the recognition of HF symptoms and patients were asked to seek medical assistance according to the signs and symptoms on the magnet. The magnet addressed changes in heart failure symptoms: green color was goal, yellow was warning zone with weight gain, edema, and so forth, and red for go to the emergency department. It also included the telephone numbers of a 24 hour/7 days a week to the nurse telecare line if patients had questions or concerns about their HF symptoms. The intensive education provided was delivered to inform the patient of changes in symptoms that should be reported to their provider. Patients in the usual care group did not receive the magnet or enhanced educational materials. The RN-PCFs also worked on improving the patient follow-up after discharge with a goal of having them receive a primary care appointment within 5–10 days of their discharge. Intervened patients not only received increased care coordination during their hospitalization but also were referred to other VA services that benefit HF patients. The HF-specific clinic, home telemonitoring, nutrition classes, and closed circuit TV HF education were made available to intervention patients. It is estimated that the RN-PCFs spent about 45 minutes for all encounters per patient in the intervention group prior to hospital discharge while those in the usual care had 20 minutes of nurse education.

### 2.5. Data Collection

Within 48–96 hours after discharge, all study patients were called by the RN-PCFs and asked a set of questions to identify the patient's self-management of HF using a standardized script. The RN-PRCs were making similar calls prior to the study and were trained on how to make the calls and interact with the patients during an orientation program. The teach-back method was used for patients in the intervened group and education on weight management, diet, and medications was reinforced during the call. The nonintervened group was asked the same set of questions but no further education was given.

The questionnaire can be found in the Appendix. The questions evaluated the HF education that patients received while in the hospital. Patients were queried as to whether or not follow-up visits/appointments were arranged, the consistency of weight and blood pressure measurements, a recall of their diet since discharge, and changes in medication usage and provided a return demonstration of HF symptom identification. The phone interviews took about 40 minutes, on average. During the consent process, patients were asked what time of day and day of week they wanted to receive the follow-up phone call and the staff tried to reach the patient at that specified time. If the staff were unsuccessful in reaching the patient by telephone on three separate occasions the patient was excluded from the study. Additional data collection included a review of the patients' electronic medical records to obtain baseline demographic and clinical values.

### 2.6. Data Analysis

The responses from the postdischarge phone call were compared between the intervened and usual care group. Descriptive statistics were used including mean and standard deviation for continuous variables, and frequency and percentage for categorical variables. Fisher's exact test was used to compare proportions for categorical variables. Unpaired *t*-tests were used to determine the mean, standard deviation (SD), and *P* value of continuous variables. A *P* value of <0.05 was used to determine the statistical significance. Data were analyzed using SAS (Statistical Analysis Software, SAS 9.2 Inc., Cary, NC).

### 2.7. Protection of Human Subjects

Institutional Review Board (IRB) approval was obtained prior to initiating the study.

## 3. Results

### 3.1. Study Sample

**Figure 1:**
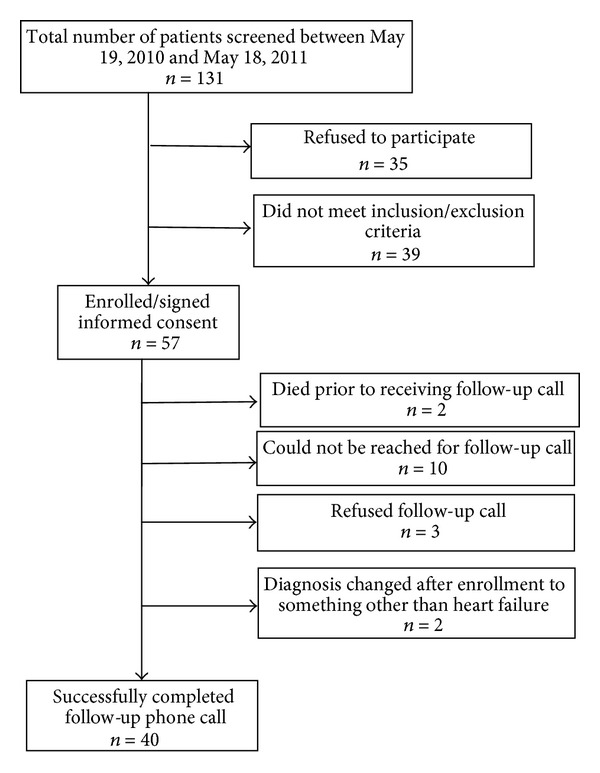


**Figure 2:**
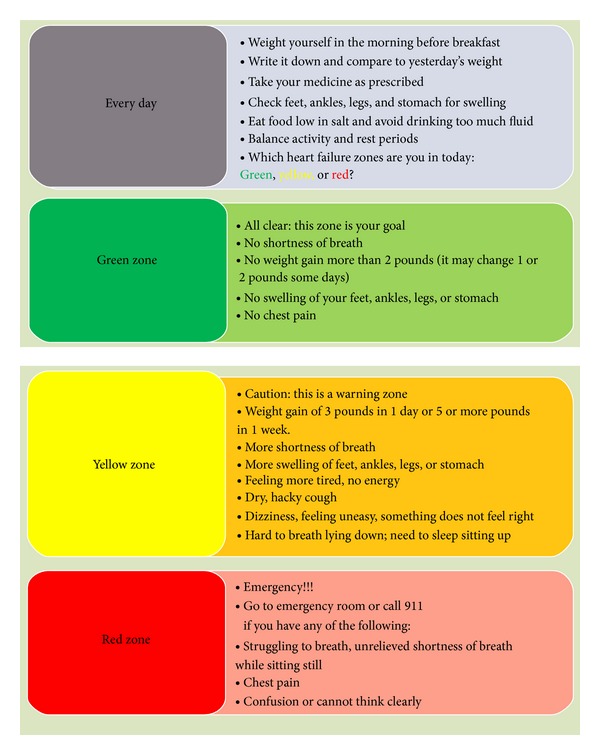
Heart failure magnet.

**Figure 3:**
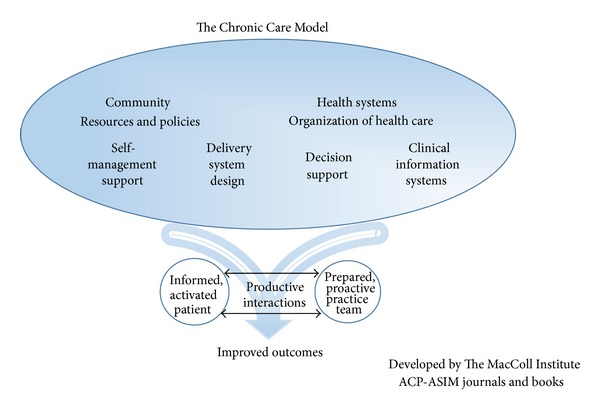
Chronic Care Model.

Between May 2010 and May 2011 there were 131 people screened for enrollment in this study (Figure 1). Of these 131 patients, 35 patients refused to participate in the study and 39 did not meet inclusion/exclusion criteria, leaving 57 patients that were enrolled and signed a consent form. Of these 57, 40 (20 intervened and 20 nonintervened) successfully completed the discharge phone call questionnaire. Patients were excluded from the study if they did not meet the inclusion criteria as stated in the research protocol. Patients were also excluded if they were approached to enroll in the study and refused to participate. The 17 patients excluded after informed consent were due to the following reasons: 2 died prior to receiving the follow-up phone call, 10 could not be reached for the follow-up phone call, 3 refused the follow-up call, and 2 had a change to their diagnosis other than heart failure after being enrolled in the study. After the patient was admitted, the patient's managing team provided follow-up during the course of stay and may have decided not to confirm the admitting team's diagnosis. Two patients were excluded because the final diagnosis was not heart failure.

The study sample ranged in age from 41 to 86 years old, with a mean age of 67 years (SD = 9.51). Ninety-eight percent (98%) were male, 75% were white, 83% were non-Hispanic, and 60.0% were ever smokers (10% current and 50% former smokers). There were no significant differences between study groups with regard to the baseline data, presented in Table 2. Also, no significant differences were detected between study groups and the presence of comorbid conditions such as renal diseases, chronic obstructive pulmonary disease, diabetes mellitus, atrial fibrillation, hypertension, stroke, coronary artery disease, and anemia.

Discharge phone call questions included information on weight, blood pressure (BP), diet and fluid restrictions, medications, HF symptoms and signs, and follow-up appointments with a primary care team or cardiologist. The results can be found in Table 3. Those in the intervention group were more likely to have a scale at home (*P* value = 0.004, OR = 1.73, 95% CI = 1.15, 2.6), weigh self every day (*P* value ≤0.0001, OR = 3.16, 95% CI = 1.60, 6.22), and write down daily weight (*P* value ≤0.0001, OR = 2.57, 95% CI = 1.39, 4.76) to practice different or new health behaviors (*P* value = 0.029, OR = 1.61, 95% CI = 1.04, 2.50). Other discharge phone call responses were not statistically significant. We also looked at 90-day heart-failure specific readmissions. There were 4 patients in the intervened group that were readmitted and 6 in the nonintervened group (*P* = 0.72). Additionally, we found that 4 patients in the nonintervened group had an emergency department visit for heart failure exacerbation 30 days after discharge versus 2 in the intervened group (*P* = 0.66).

## 4. Discussion

The objective of this study was to examine whether intensive education and care coordination would increase self-management in patients with HF. The changes in clinical practice and care coordination that were made in this study were associated with HF patients better able to self-manage their weight, a key factor in the reduction of HF rehospitalizations. Patients who received the intervention were given specialized education on how to monitor their weight and to look out for warning signs that would prevent an emergency room visit or hospitalization.

These findings suggest an improvement in understanding of HF symptoms in the patient and further reinforcement of this knowledge could lead to reductions in the number of hospitalizations or severity of HF incidents. In this pilot study, we found that the intervened group had fewer rehospitalizations and fewer emergency room visits associated with heart failure symptoms, although the difference was not statistically significant. With a larger sample size the difference in effect sizes may have been bigger.

This study had limitations that may alter its validity. The use of a follow-up phone call to assess one's newly acquired knowledge of HF self-management may fail to capture the true outcome of our study. While great effort was made to ensure a safe transition home from hospital care there is no objective way to measure the stress that afflicts a patient during this time and may bias the way the patients responded to the questionnaire. There may have also been recall bias, as the patient had to recall some of the information asked of them during the postdischarge interview. Also, vigorous attempts were made to identify the universe of HF patients to reduce sampling bias. Some HF patients with atypical presentation and multimorbid conditions that mimic some HF signs and symptoms (COPD and pneumonia) may have been excluded from the study. Additionally, despite verification with the patient during enrollment of phone number to reach patients after discharge and willingness of the investigator team to conduct postdischarge phone calls after regular business hours and weekends, many patients were not reachable by phone. Another potential bias in the study was the use of the investigator-designed questionnaire administered. The reliability and reproducibility of the tool were not assessed prior to its use.

This study was not a randomized controlled trial, and there could have been underlying differences between the two groups that could have influenced the outcomes of the study. Possible confounders may be differences between study groups in underlying disease conditions such as hypertension, coronary artery disease, diabetes mellitus, and atrial fibrillation to name a few. However, when assessing baseline characteristics between groups, there were no reported differences between baseline characteristics. Moreover, patients could have been in either the intervened or nonintervened groups based on the random rotation of the medicine team during the patient's admission to the hospital.

Another limitation to note is the population examined. When working with a specific population such as veterans one must take into consideration the differences existing in this group compared to the population as a whole. Veterans face different challenges than the general population, such as disproportionate rates of homelessness, with approximately 16% of all homeless adults being veterans [26], and also having high levels of mental illness [27]. Thus the generalizability of these findings may be limited to this particular subpopulation. This is not to say that these findings are insignificant given the sizeable population of veterans in the United State. Overall, this pilot study has demonstrated improvements in self-management of HF among veterans. The foundation has been built for further interventions as continued improvement in care and education aims to reduce HF readmissions. Future research will examine over time the association of HF events in one year after discharge. These future studies are being planned and several funding agencies are interested in this type of research. Findings from this study will help design randomized controlled trials to provide more robust indicators on the efficacy of the treatment. Additionally, future work will involve reporting on the qualitative and behavioral elements that characterize the sample.

## Figures and Tables

**Table 1:** Usual care versus intervention.

Usual care	Intervention
Disease-specific information provided in two-page HF handouts.	HF information about incidence, prognosis, sodium restriction hints, links to HF organizations, list of HF-related resources at the facility.

Staff verify date/time of follow-up appointment.	Verifying: whether transportation was available at the scheduled appointment, if each prescription was obtained at discharge, and what questions the provider will ask at followup.

Staff confirm the patient knows the phone number of his/her outpatient clinic.	Provided nurse facilitator name/phone as well as clinic name and phone

Follow-up phone call asks general question: “Are you doing OK?” “Did you pick up your prescriptions?”	A set of HF-specific questions is asked in conversation on follow-up phone call, carefully constructed to avoid questions that lead to an automatic “Yes” or “No” from the patient.

Inpatient staff typically work 12-hour shifts; during a 3-4 day stay, continuity of staff is unlikely. This makes information exchange inconsistent, repetitious, or overlooked.	Nurse facilitator was present Monday through Friday; if not, specific nurse replacement's name was given to the patient.

Time interval for postdischarge appointment is not set by policy; date/time is generated according to open vacancies.	Study obtained administrative policy support to allow nurse facilitator to require postdischarge followup within five to ten days, even if provider had to be overbooked.

No teach-back method used. Understanding of what patient learned not confirmed.	Teach-back method used. Patient explained the educational information received back to the practitioner.

Staff nurses spend approximately 20 minutes per encounter per patient prior to discharge.	Nurse facilitators spend approximately 45 minutes per encounter per patient prior to discharge.

**Table 2:** Baseline study participant data.

Baseline characteristics	Intervention group (*n* = 20)	Standard care group (*n* = 20)	*P* value
Age, mean (SD)	64.65 (20)	68.45 (20)	0.21
Male, % (*n*)	95.00 (19)	100.0 (20)	1.00
White, % (*n*)	75.00 (15)	75.00 (15)	1.00
African American, % (*n*)	0.00 (0)	10.00 (2)	0.49
Race, other, % (*n*)	25.00 (5)	15.00 (3)	0.69
Non-Hispanic, % (*n*)	75.00 (15)	90.00 (18)	0.41
Married, % (*n*)	50.00 (10)	45.00 (9)	0.75
Ever smokers, % (*n*)	60.00 (12)	70.00 (14)	0.51
Primary care appointment within 14 days of discharge, %, *n*	50.00 (10)	45.00 (9)	1.00
Troponin (TNI), mean (SD)	0.04 (0.03)	0.07 (0.09)	0.168
Creatinine, mean (SD)	1.31 (0.63)	1.21 (0.40)	0.551
Brain type natriuretic peptide (BNP), mean (SD)	908.42 (900.42)	765.97 (706.33)	0.581
Sodium (NA), mean (SD)	138.90 (3.99)	140.60 (3.38)	0.154
Hemoglobin (HGB), mean (SD)	12.53 (1.96)	12.27 (1.67)	0.655
Ejection fraction (EF), mean (SD)	34.42 (16.47)	29.75 (12.88)	0.325
Heart rate, mean (SD)	78.35 (21.09)	88.85 (23.61)	0.146
Systolic blood pressure (SBP), mean (SD)	136.75 (26.06)	124.25 (19.16)	0.093
Body mass index (BMI) (kg/m^2^), mean (SD)	31.48 (8.57)	33.12 (8.80)	0.555

**Table 3:** Result of postdischarge phone call questions relative to study group.

Follow-up phone call outcomes	Intervention group % yes (*n*)	Standard care group % yes (*n*)	*P* value
Patient has a follow-up appointment scheduled	85.00 (17)	85.00 (17)	1.000
Patient has scale at home*	95.00 (19)	55.00 (11)	0.004
Patient weighs self every day*	95.00 (19)	30.00 (6)	<0.0001
Patient writes down weight*	90.00 (18)	35.00 (7)	<0.0001
Patient is watching BP since discharge	75.00 (15)	70.00 (14)	0.723
Patient has BP cuff at home	80.00 (16)	85.00 (17)	1.000
Patient checks BP every day	65.00 (13)	60.00 (12)	0.744
Patient told by VA to restrict diet	100.00 (20)	85.00 (17)	0.231
Patient told by VA to restrict fluids	80.00 (16)	65.00 (13)	0.288
Patient understands consequences of high sodium diet	78.95 (15)	65.00 (13)	0.480
Patient has all the medications	95.00 (19)	100.00 (19)	1.000
Does patient recall HF symptoms to watch out for	95.00 (19)	90.00 (18)	1.000
Patient has understanding of what to do when HF symptoms present	85.00 (17)	80.00 (16)	1.000
Patient knows who to call if HF symptoms present	100.00 (20)	85.00 (17)	0.231
Patient is practicing different/new health behaviors*	89.47 (17)	55.56 (10)	0.029

*Postdischarge phone call questions that were significantly different (*P* value <0.05) between groups.

## Appendix

### Discharge Phone Call Questions

*QUERI-CHF Follow-Up Phone Interview Questions.*

I want to find out from you how have been doing in terms of your heart failure since you were sent home from the hospital—number of days ago. So, I am going to ask you some questions, please just answer the best you can.

1. First off, have you scheduled a follow-up appointment with your doctor?
  - Who is your appointment with?
  - Who made the appointment?
  - If so, when is the appointment?
  - How are you getting to the appointment?
2. When you were in the hospital for heart failure, do you remember who talked  with you about the things you need to know in order to take care of yourself and your heart?
  - If so, can you tell me a few things you were told? Listen to what the patient says and follow up by asking the patient to explain. Then, ask the patient the other questions related to what they may not have mentioned.
3. Tell me how you have been watching your weight since you have been back at home/out of the hospital.
4. Depending on answer…Ask the following:
  (a) Do you have a scale at home? Did the VA send you home with a scale?
  (b) Do you weigh yourself every day? Do you write it down? Why is it a good idea to write your weight down every day?
  (c) Why is it important to watch your weight? What does it mean to you when you start to gain weight all of a sudden? What can cause you to gain weight?
  (d) What can/should you do if that happens?
5. Have you been watching your blood pressure since you have been back at home/out of the hospital?
  - Do you have a BP cuff at home?
  - Do you check your BP every day?
  - Do you write down your BP or send it to the hospital?
  - Why is it important to watch your BP?
  - What does it mean to you when your BP gets high all of a sudden? What can cause your BP to go up?
  - What can/should you do when that happens?
6. Tell me about what you have been eating (your diet) since you have been back at home/out of the hospital?
  - Were you told at the hospital that you should restrict your diet (water, salt)?
  - Were you told at the hospital that you should limit the fluids you drink every day? Depending on patient, ask about specific dietary habits in last 48 hours.
7. For example:
  (c) What did you eat today for breakfast/lunch? What did you have to drink today?
  (d) Tell me about eating foods that have lots of salt in them.
  (e) Why is it important to limit liquids/certain foods?
8. Tell me about the medications you are taking for your heart failure?
  (a) How are you taking your water pill? (or other critical medication)?
  (b) How many times a day do you take that pill? (Ask for each pill)
  (c) Do you have all the medications that you or your doctor thinks you need? Do you need any refills?
9. Who do you live with?
10. Do you have family or friends who help you with any of the following? Who?
  - Taking your medications
  - Taking your BP
  - Weighing yourself and writing down your weight?
  - Going to the grocery store/chores?
  - Cooking
  - Transportation
11. Can you tell me a few symptoms to watch out for in order to avoid heart failure or going back to the hospital for the treatment of your heart failure?
  - What should you do when you have any one of these symptoms?
  - Who should you call?
  - What zone are you in right now? Green, Yellow, or Red?
12. What was the most important thing you learned in the hospital about heart failure?
13. Are you doing anything different for your health now, compared to before you were in the hospital, for heart failure?
14. What other VA services are you receiving to help you with your heart failure? NONE (Home Tel-Health (with the machines at home), Cardiac Rehab, PT, CHF Clinic, Home Health Visits)
15. Are you receiving any treatments outside the VA to help you with your heart failure?
16. Can you tell us something the VA could do to help you manage your heart failure?
17. Do you have any questions for us?

## Abbreviations

HF: Heart failure
AHA: American Heart Association
PCF: RN patient care facilitator
SD: Standard deviation
BP: Blood pressure
VHA: Veterans Health Administration
RCT: Randomized controlled trial
LV: Left ventricular.
